# West Meets East in a New Two-Polarities Model of Personality: Combining Self-Relatedness Structure With Independent-Interdependent Functions

**DOI:** 10.3389/fpsyg.2021.739900

**Published:** 2021-12-17

**Authors:** Weiqiao Fan, Mengting Li, Frederick Leong, Mingjie Zhou

**Affiliations:** ^1^Research Institute for International and Comparative Education and Department of Psychology, Shanghai Normal University, Shanghai, China; ^2^Faculty of Education, The University of Hong Kong, Pokfulam, Hong Kong SAR, China; ^3^Department of Psychology, Michigan State University, East Lansing, MI, United States; ^4^Institute of Psychology, Chinese Academy of Sciences (CAS), Beijing, China

**Keywords:** personality, self, relatedness, structure, function, west, east

## Abstract

Self and relatedness are the two most essential dimensions of personality, as indicated in many personality theories, and have been supported by numerous empirical studies conducted in the western (individualistic) and eastern (collectivist) contexts. However, because of a confusion or failure to distinguish the structure and function of personality, popular theories (e.g., the Big Five model) do not make logic distinctions between these two basic personality dimensions. In terms of the cultural-relevant feature, both self and relatedness and their specific aspects may be variously highlighted in different cultural settings. On the basis of a re-examination of several crucial two-dimension (namely, self and relatedness) personality theories derived from the east and west, we reconstruct a new two polarities personality model to include not only self and relatedness but also the independent and interdependent functions in terms of some popular personality theories from western and eastern cultures. Theoretically and empirically, self and relatedness should be the basic structures of personality, whereas independence and interdependence should be the basic functions of personality. Self and relatedness have independent and interdependent functions; however, due to the cultural relevance of personality, the functions should be variously emphasized in different contexts. Several possible future research directions are discussed.

## Introduction

As mainstream of personality psychology (e.g., Eysenck, 1970; McCrae and Costa, 1989; Ashton et al., 2009), most western-derived models have been strongly concerned with intrapsychic dimensions and the lives of people as individuals—such as their occupation, marriage status, family, and age. On the other hand, a systematic analysis of personality with sufficient attention to interpersonal dimensions is seriously lacking (Freedman et al., 1951; Leary, 1957; Wiggins, 1979; Yang, 2006; Blatt, 2008; Cheung and Ho, 2018).

With societies becoming more multicultural and more individuals crossing multicultural identity boundaries, personality psychology must “move beyond the critiques of imperialism and nationalism to a level of international cooperation with greater cultural sensitivities” (Cheung et al., 2011, p. 600). In recent decades, some researchers have attempted to describe personality from a comprehensive perspective in a global context. Particularly, in light of Hofstede’s (2001) cultural framework with western (individualistic) or eastern (collectivistic) orientation, both self and relatedness, as two most essential dimensions of personality, have been highlighted in those personality models derived from both western (individualistic) and eastern (collectivistic) cultures (e.g., Cheung et al., 1996; Yang, 2006; Blatt, 2008). Due to the differences between eastern and western cultures these binary personality theories derived from the east or the west showed some unique characteristics of self and relatedness, which have led to certain differences in understanding personality by scholars from east and west.

Particularly, because of greater emphasis on the individualistic and independent nature of western culture, a few interdependent-related characteristics (e.g., agreeableness and parts of sub-dimensions of extraversion–warmth and gregariousness) have been included in some popular models (e.g., McCrae and Costa, 1989; Graziano and Tobin, 2009), but relatedness-relevant personality dimensions have not been logically or structurally (e.g., Big five model and Erikson’s model) in or have often been slightly overlooked in those mainstream theories and assessments of western personality (e.g., Cheung et al., 2003). Furthermore, it is unclear whether the “self” (or some related terms such as autonomy, agency, introjection, and individual orientation) and “relatedness” (or some other related terms such as communion, sociotropy, anaclitic, and social orientation) have equivalent meanings in models derived from eastern and western settings or some different aspects of “self” and “relatedness” have been variously emphasized in different backgrounds.

Is it possible that self or relatedness possess cultural-specific representation or different functions for individuals in different cultural contexts? Should or can both self and relatedness be further deconstructed under the construct of personality? Accordingly, we may need a new construction for the dualistic theoretical framework that can cover “self and relatedness” and at the same time accommodate the differences of functions in personality between the east and the west (i.e., “independent” and “interdependent” personality functions).

Based on the review of those important two-dimension models derived from west and east, we will argue that there are no fundamental differences in structure (both self and relatedness) and functions (both independent and interdependent) between eastern and western personality. The core difference may lie in the degrees of emphasis on different structure content in each culture, which is culturally represented by specific personality function expression. Therefore, a new two-polarities model that criss-crosses structural and functional aspects of personality is proposed. We reconstruct the dualistic personality model that includes not only self and relatedness but also independent and interdependent functions. We advance a new two polarities model comprising four sub-dimensions of personality: independent self, interdependent self, intrapersonal relatedness and interpersonal relatedness. We deconstruct the existing typical personality models by eastern and western personality psychologists in light of our new two-polarities model (see Table 1 for a diagrammatical representation). We expect our work to increase the understanding of personality structure and function from an enlarged perspective that incorporates both eastern and western cultures.

**TABLE 1:** Personality structure under the framework of self and relatedness with the function of independence and interdependence.

Function Structure	Self	Relatedness
Independence	Independent self: Autonomy (Angyal, 1951; Erikson, 1968; Ryan and Deci, 2000), agency (Bakan, 1966; Pincus, 2005); achievement (McAdams, 1985; McClelland, 1985), industry (Erikson, 1968), introjective (Blatt, 2008), neuroticism (McCrae and Costa, 1989), openness (McCrae and Costa, 1989), extroversion (referring to the sub-dimensions of assertiveness, activity, excitement seeking, and positive emotions; McCrae and Costa, 1989)	Intrapersonal relatedness: Agreeableness (McCrae and Costa, 1989), extroversion (referring to the sub-dimensions of warmth and gregariousness; McCrae and Costa, 1989), trust (vs. mistrust) (Erikson, 1968), discipline, graciousness, thrift, traditionalism, defensiveness, and veraciousness (Cheung et al., 2013)
Interdependence	Interdepedent self: Conscientiousness (McCrae and Costa, 1989), need of power (Winter, 1973), initiative (Erikson, 1968), Face (Cheung et al., 2013; Zhai, 2013), *Lian* (Zhai, 2013)	Interpersonal relatedness: Affiliation (McAdams, 1985; McClelland, 1985), anaclitic (Blatt, 2008), cooperation (Freedman et al., 1951; Noam and Fischer, 1996), communication (Bakan, 1966), intimacy (Erikson, 1968; McAdams, 1985; McClelland, 1985), socitropy (Clark and Beck, 1999); Surrender (Angyal, 1951), *Renqing*, social sensitivity, interpersonal tolerance, and harmony (Cheung et al., 2013)

*Only some typical personality dimensions in common personality models are exampled in this table.*

## Traditional Bi-Dimensional Models of Personality

### Western Models

Traditionally, two typical personality dimensions, self and relatedness, have been central in personality theories across various psychology domains, ranging from cross-cultural psychology to social psychology and psychoanalysis (Blatt, 2008; Luyten and Blatt, 2013). Developed on the basis of the perspectives of different disciplines or methodologies, the various personality theories refer to the two dimensions as surrender and autonomy (Angyal, 1951), communion and agency (Bakan, 1966; also see Pincus, 2005), sociotropy and autonomy (Beck, 1999), togetherness and individuality (Bowen, 1966), attachment and separation (Bowlby, 1969), individuation and attachment (Franz and White, 1991), affiliation (or intimacy) and achievement (or power; McAdams, 1985), mutualistic and individualistic urges (Slavin and Kriegman, 1992), individual and group identities or self and social identities (Tajfel, 1978; Turner et al., 1987), and relatedness and autonomy or competence (Ryan and Deci, 2000).

As indicated by authors such as Mikulincer and Shaver (2007) and Pincus (2005), the dialectic interaction between issues of relatedness and self in personality and personality development has been emphasized in these bi-dimensional models of personality. According to these models in western personality psychology, personality structure has a clear binary framework (for reviews, also see Luyten and Blatt, 2013). Several pivotal two-polarities models derived from western backgrounds are outlined below.

#### Interpersonal Models

In addition to primarily focusing on intrapersonal dimensions in western mainstream personality psychology, some personality scholars (e.g., Freedman et al., 1951; Leary, 1957; Wiggins, 1979; Kiesler, 1996; Pincus, 2005) have highlighted interpersonal attributes and suggested that two orthogonal dimensions underlie interpersonal traits, attitudes, and behavior in both normal and disrupted personality development: agency (or social dominance) and communion (or nurturance or affiliation). Conceptually, agency is clearly related to the self-definition (autonomy) dimension, whereas communion is congruent with the relatedness/sociotropic dimension. The interpersonal models assume that normal personality development involves a balance between agency and affiliation (Laforge et al., 1954; Wiggins, 2003; Pincus, 2005). The two-factor model can be arranged in a circumplex model comprising four quadrants (Freedman et al., 1951; Leary, 1957), and this model is empirically supported in the literature of western personality. For example, studies have demonstrated that anaclitic or sociotropic individuals are located in the friendly-submissive quadrant, evidencing high levels of dependency and low levels of dominance, whereas introjective or autonomous individuals exhibit the opposite pattern, being located in the hostile-domineering quadrant (e.g., Pincus, 2005; Ravitz et al., 2008).

#### Blatt’s Two-Polarities Model

Blatt and colleagues (Blatt and Blass, 1996; Blatt, 2008; Luyten and Blatt, 2011, 2013) have argued that personality develops through a complex dialectic transaction between two fundamental psychological developmental dimensions: interpersonal relatedness—the development of increasingly mature, intimate, mutually satisfying, and reciprocal interpersonal relationships—and self-definition—the development of an increasingly differentiated, integrated, realistic, and essentially positive sense of self or identity. This model further emphasizes the importance of interpersonal relationships on the basis of focusing on the self-construction of personality. Blatt (2008) argued that interpersonal relatedness and self-definition, two fundamental developmental processes, evolve through a life-long dialectic transaction such that progress in relatedness (anaclitic) or self-definition (introjective) development facilitates progress in the other. The two main lines of development and the two personality dimensions are independent but also promote each other. For example, meaningful and satisfying relationships may contribute to self-construction, and a defined self may lead, in turn, to more mature levels of interpersonal relatedness (Luyten and Blatt, 2011, 2013).

#### Beck’s Cognitive Behavioral Model of Personality

Like in the aforementioned work in the western field of personality, Beck (1983, 1999) defined two central dimensions for deconstructing personality and emphasized the interpersonal aspect of personality as well as the intrapersonal aspect. Beck’s model highlights a favorable balance between autonomy and sociotropy as the hallmark of adaptive personality functioning. According to Beck (1983, p. 273), sociotropy (or social dependency) reflects “the person’s investment in positive interchange with other people…including passive-receptive wishes (acceptance, intimacy, understanding, support, guidance).” Sociotropic individuals care particularly about other people’s attitude toward them, and they often try to please others and maintain their attachments (Robins and Block, 1988). By contrast, autonomy (or individuality) reflects “the person’s investment in preserving and increasing his independence, mobility, and personal rights; freedom of choice, action, and expression; protection of his domain…and attaining meaningful goals” (Beck, 1983, p. 272). Autonomous, achievement-oriented individuals are mainly concerned about the possibility of personal success and often try to maximize their control over the environment to reduce their probability of failure.

The distinction between the anaclitic/sociotropic/relatedness and introjective/autonomous/self-definition personality dimensions has been widely validated in both clinical and non-clinical samples (Clark and Beck, 1999; Matsumoto, 1999; Zuroff et al., 2004; Blatt, 2008). These models have also been conceptually and empirically linked to contemporary interpersonal approaches (Freedman et al., 1951; Wiggins, 1991, 2003; Pincus, 2005; Ravitz et al., 2008), attachment theory (Sibley, 2007), and self-determination theory (Shahar et al., 2006). Empirical investigations have indicated consistent differences in current and early life experiences (Blatt, 2008), and basic character and relational style (Zuroff et al., 2004) associated with these two dimensions. In addition, the three two-dimension models overlap to a certain extent.

Recently, Luyten and Blatt (2013) broadly reviewed empirical evidence supporting the two-dimension model and concerning the neurobiological and evolutionary foundations (e.g., Beebe et al., 2007; Simeon et al., 2011). Luyten and Blatt (2013) also reported the effects of developmental factors, gender, and sociocultural issues on the development of interpersonal relatedness and self-definition (e.g., Fraley and Roberts, 2005; DiBartolo and Rendón, 2012). The two polarities model provides theoretical and empirical utility concerning normal and disrupted personality and its development, which is largely influenced by evolutionary, biological, and sociocultural factors and their interactions.

Although different theoretical labels are used in these various theories, there is remarkable theoretical and empirical overlap. Moreover, emerging evidence indicates that these theories, which have been developed within differing theoretical approaches, assess aspects of the two fundamental dimensions (relatedness and self) at different levels of abstraction, indicating that the extant two-dimensional models of personality organization and development can be hierarchically organized (Sibley, 2007; Sibley and Overall, 2007; Luyten and Blatt, 2013). However, the dualistic structure of personality has historically been slightly overlooked in the mainstream of personality psychology.

### Eastern Models

In their experiences of personality research and applications, some east scholars (e.g., F. M. Cheung, K. Yang, and their colleagues) found that western-based mainstream personality inventories (e.g., the Chinese Minnesota Multiphasic Personality Inventory; Cheung et al., 1992) could not provide a reliable and valid assessment of Chinese individuals’ personality. Therefore, in response the challenges to Chinese personality in theory and application, eastern psychologists highlighted the dimension of relatedness as a supplement to western individualistic models (e.g., Cheung et al., 1996; Yang, 2006).

Two individual- and relational-oriented models are briefly reviewed in this section. The first is a four-factor model of personality assessed using the Cross-Cultural (Chinese) Personality Inventory (CPAI) developed by Cheung et al. (1996; 2003; 2013). The other is a four-dimension model of personality proposed by Yang (2006). These two models developed from eastern backgrounds largely reflect a binary framework for understanding personality traits with individual and relational orientations across cultures.

#### Cheung’s Binary Personality Model Measured Using the Cross-Cultural (Chinese) Personality Inventory

Since the early 1990s, they developed various CPAI measurements, including the adult version (CPAI), the revised version (CPAI-2), and the adolescent version (CPAI-A) (Cheung et al., 2008b, 2013; Fan et al., 2008) with a combined emic–etic approach (Cheung et al., 2011). In the personality model assessed using the CPAI inventories, a Chinese indigenous personality dimension, interpersonal relatedness, is measured. This dimension evaluates the characteristics associated with the relationships between people (with society, family, and relatives) in the personality structure. Interpersonal relatedness reflects “a strong orientation toward instrumental relationships; emphasis on occupying one’s proper place and engaging in appropriate action; avoidance of internal, external, and interpersonal conflict; and adherence to norms and traditions” (Cheung et al., 2001).

Interpersonal relatedness encompasses not only the connotations of an individual’s intrinsic characteristics related to interpersonal communication and the subjective attitude toward relationships with people (related sub-dimensions such as discipline, graciousness, traditionalism, thrift), but also the external behavior shown in daily interpersonal communication (related sub-dimensions including *renqing*, social sensitivity, interpersonal tolerance, and harmony). Specifically, for example, graciousness measures how kind and broad-minded people are in their dealings with others. One item of graciousness is “When someone offends me, I will always bear that in mind (reversed).” *Renqing* measures the individual’s adherence to cultural norms regarding reciprocal interactions such as courtesy rituals, exchanging resources, maintaining and utilizing useful ties, and nepotism. For example, one item of *renqing* is “If a friend or relative was hospitalized, I would definitely go to visit him/her.”

The rest three factors in the CPAI model assessed using the CPAI measurements are largely correlated with Big Five factors, which mainly reflect an individual or intrapersonal orientation (Cheung et al., 2001, 2003, 2008a). For example, the social potency/expansiveness factor in the CPAI-2 and CPAI-A evaluates the personality traits of individuals pursuing change, innovation, self-development, and the realization of individual values, which are largely related to openness and extraversion in the Big Five. The core meaning of emotional stability in the CPAI-A lies in the emotional stability and adjustment of self-cognition and attitude; emotional stability is partially related to neuroticism within the Big Five. The core meaning of dependability in the CPAI-2 and CPAI-A is evaluation of reliability, seriousness, and responsibility, and dependability is strongly related to sense of responsibility and neuroticism within the Big Five. In addition, accommodation in the CPAI-2 mainly assesses an individual’s attitude toward society or others and may reflect intrapersonal relatedness or social cognition—how a person relates to society or others.

Subsequent cross-cultural research has suggested that the CPAI personality framework is relevant across cultures and that the constructs derived from an eastern context—interpersonal relatedness–related personality constructs (e.g., *renqing*, harmony, social sensitivity, family orientation, and traditionalism)—are not restricted to the Chinese context and can be validated in some western cultures (Cheung et al., 2001, 2003, 2006; Lin and Church, 2004; Born and Jooren, 2009; Iliescu, 2009; Fan et al., 2011, 2012). For example, Lin and Church (2004) discovered that the interpersonal relatedness factor was well supported in both Chinese American and European American samples; moreover, the scores of European Americans on family orientation, which is highly valued in traditional Chinese culture, were significantly higher than those of Chinese Americans. Born and Jooren (2009) surveyed Dutch university students and Iliescu (2009) surveyed a Romanian sample, and found that the CPAI-2′s four-factor structure was largely supported.

Therefore, the personality model assessed by the CPAI (including the updated version CPAI-2 and the CPAI-A) largely reveals two types of personality factor. One type is largely related to or overlaps with some factors in the Big Five model, which was originally derived from western cultures and mainly reflects the intrapsychic dimensions of personality traits highlighted in those cultures. The other type is interpersonal relatedness as defined by Cheung and her colleagues; for this type, a group of interpsychic dimensions of personality traits highlighted in Chinese and most eastern cultures is assessed.

#### Yang’s Binary Personality Model in Terms of a Four-Level Personality Framework

Yang (2006) also developed a four-level conceptual scheme for classifying personality traits under a dual high-order personality structure composed of individual-oriented personality attributes and social-oriented personality attributes, which comprise relational-oriented, group-oriented, and other-oriented attributes. Therefore, Yang actually proposed a binary model for understanding personality on the basis of both dispositional and cultural psychological approaches (Church, 2000).

According to Yang (1995, 2006), a person’s aptitudes, temperament, needs, cognitions, affect, and behaviors, which are relatively enduring characteristics, together form the person’s personality, which results from a particular ecological, social, cultural, and historical milieu. Culture and personality attributes (even aptitudes and temperament) are assumed to be more or less bidirectionally determined and mutually constituted (Markus and Kitayama, 1998). In a specific living environment, a person’s interactions with their personal self (similar to the construct of the independent self proposed by Markus and Kitayama, 1991) construct individual-oriented attributes such as autonomy, independence, agency, and competence in both eastern and western contexts. An individual’s interactions with another person may help form relationship-oriented attributes such as harmony and *renqing* (Cheung et al., 1996, 2013). A person’s interactions with their family and other groups may yield group-oriented attributes such as family orientation (Fan et al., 2014) and leadership. Finally, an individual’s interactions with real or imagined non-specific unidentified others as the generalized audience in the social environment may form other-oriented attributes such as face (i.e., *mianzi* in Chinese, Zhai, 2013) and defensiveness (Cheung et al., 1996, 2013).

These four levels of personality traits are composed of a person’s personality structure for people of all cultures, but different dimensions are given differing importance depending on the cultural background of individualism or collectivism. In fact, personality attributes within individualist and collectivist societies may have deep social and cultural explanations. In eastern societies (e.g., China and Japan), people are inclined to comply with social roles, norms, obligations, customs, and practices, and the relational-oriented self acts as the major anchoring and stabilizing center for consistent and coherent personality functioning in everyday life. In western societies (e.g., the United States), people are inclined to assert the self and appreciate their differences from others. The individual-oriented self acts as the major anchoring and stabilizing center for consistent and coherent personality functioning in everyday life, whereas sociocultural factors may be readily changed to suit the person’s needs.

In eastern societies, relationship-oriented, group-oriented, and other-oriented attributes, which have stronger connections with people’s daily life than in western societies, are especially prevalent. However, even people in social-oriented societies (e.g., China) may have certain individual-oriented characteristics in some circumstances (Markus and Kitayama, 1991). For example, when people attend a banquet, they sometimes dress informally and even to stand out. This may reflect a certain individualistic orientation. In western societies, individual-oriented attributes, which have stronger connections with daily life than in eastern societies, are especially prevalent. However, this does not mean that people in individual-oriented societies do not exhibit social-oriented characteristics in some circumstances (e.g., in religious groups and some small towns and rural communities; Bellah et al., 1985; Markus and Kitayama, 1991). For example, in western societies, when people attend a banquet, they often dress formally, which may reflect a certain collectivist orientation.

Therefore, the four levels of personality traits define a dualistic framework of personality comprising aspects of self-construal in personality and aspects of a broader understanding of relatedness–construal in personality in terms of macro and micro societies. Therefore, both Cheung and Yang have separately defined a bi-dimensional structure of personality on the basis of their eastern cultural backgrounds; some details still need to be further verified in theory and practice, however.

### Ways for Western Models to Meet Eastern Models

Based on the above reviews, we conclude that both western and eastern personality theorists noted that both self and relatedness are important and foundational factors of personality across east and west. This may be the reason that there are dualistic models parallel to western ones emerging in eastern culture. However, these scholars from the west and the east may have great differences in understanding this dualistic structure, and at the same time, there is a lot of space for modification in their models. In the next section, we first propose a new two-polarities model, and then deconstruct those existing dualistic personality models derived from west or east and reconstruct the structure and function of self and relatedness from a cross-cultural perspective.

## A New Two Polarities Model of Personality

As noted earlier, efforts to use a dichotomy to analyze personality have never stopped. For example, The idioms “round outside and square inside” or “sageliness within and kingliness outside” in Confucian philosophy refer to how to be a person and do things in the world (Cheung et al., 2008b; Zhou et al., 2021); they also may reflect one explanation for personality with a dialectical thinking pattern. The “inner square” or “sageliness” means that a person should behave according to certain principles and maintain their independence and integrity. This may be similar to the self in the two-polarities model of personality. The “outer round” or “kingliness” means that a person should also live in harmony with their surroundings by using certain interpersonal strategies or approaches. This may be similar to relatedness in the two-polarities model of personality. Similarly, Blatt and Blass (1996) have tried to reanalyze Erikson’s (1968) eight-stage linear developmental line, adding an additional stage—cooperation versus alienation, with the framework of self and relatedness.

This is also true for the widely recognized Big Five personality model. For example, on the basis of a series of studies supporting the Big Five factors, Digman (1997) deconstructed the Big Five model into a two-dimension higher-order factor structure; the two dimensions were labeled α (comprising agreeableness, conscientiousness, and emotional stability) and β (comprising extraversion and openness). However, because this two-factor model was mainly derived from empirical results but not theory-based, its implications in theory and practice are limited (e.g., Ashton et al., 2009). One possible reason may be that both α and β factors consist of aspects of both relatedness and self, which theoretically and practically reflect different personality structures, or functions (McCrae and Costa, 1989). Freedman et al. (1951) and Leary (1957) have also argued that a total personality consists of both structures and mechanisms.

Our review also indicates that even if both self and relatedness are largely defined as the two central contents of personality, the corresponding bi-dimensional models do not provide a logical, systematic, or consistent explanation of the two superordinate dimensions. One possible reason is that the structure (e.g., traits) and functions of those models have not been fully explained. The other may be that both self and relatedness may reflect various functions in a specific cultural setting.

### Considering Both Structure and Function of Personality

In this article, the structure of personality refers to the dualistic framework of self and relatedness; the function can be understood in terms of the utilities of the structure of personality (namely self and relatedness) in realizing the mechanism to make an individual dependent or independent (e.g., Freedman et al., 1951; Leary, 1957). Various scholarly contributions have discussed the interplay of these two polarities.

Loevinger (1976), for instance, pointed out the main function of self is to help the individual to integrate one’s life experience and adapt into one’s environment. From a cross-cultural perspective, Hashimoto and Yamagishi (2016) compared the adaptive roles of self-construal with independence and interdependence between US and Japan participants. Although there are some differences in the dimension of interdependence between US and Japan participants, the framework of a duality of independence and interdependence was empirically supported (Hashimoto and Yamagishi, 2016). This adaptation actually includes one’s autonomy and attachment (e.g., Mahler et al., 1975; also see the Erikson’s (1968) psychological development) or agency and communion (Bakan, 1966).

In traditional models of personality, structure and functions are often both considered; however, except for a few models (e.g., Freud’s and Eysenck’s frameworks), they are not constructed under any specific rationale. For example, the Big Five model has often been considered a typical trait theory of personality comprising five key personality traits; however, those traits may need to be further distinguished in terms of their different functions. For example, agreeableness and extraversion define the plane of interpersonal behavior (McCrae and Costa, 1989), but they also reflect extremely different functions in the dimension of interpersonal personality. As argued by Digman (1997) on the basis of Eysenck’s (1970) viewpoint, extraversion involves not only an interest in social interaction but also active, zestful, and venturesome activities in life and interpersonal relations; extraversion mainly achieves a function for construct an independent self (referring to the sub-dimensions of assertiveness, activity, excitement seeking, and positive emotions; McCrae and Costa, 1989). However, agreeableness describes individual differences as being likeable, pleasant, and harmonious in relations with others, and also reflects some characteristics such as kindness, warmth, and considerateness (Graziano and Tobin, 2009).

As another example, although Cheung and colleagues proposed interpersonal relatedness as an indigenous-Chinese personality factor, this factor is complex and must be deconstructed because different subordinate factors reflect either independent or interdependent functions of personality. Specifically, although both discipline and *renqing* are related to a person’s interpersonal environment, discipline indicates how independent a person is from others, and reflects a function of relatedness to make an individual more independent by one’s inner attitude toward interpersonal communication; whereas *renqing* defines how interdependent a person is with others, and mainly reflects a function of relatedness to make an individual more interdependent by one’s extra behaviors with others.

Theoretically, personality has two basic functions related to the internal and external environments. The first is to maintain independence (Loevinger, 1987), achieve ego functional autonomy (Allport, 1961), and then construct self-identity (Erikson, 1968; Pals, 2001). This independence or autonomy helps a person meet their needs for achievement and power (Murry, 1938; Maslow, 1970). The second is to connect a person with their social environment (e.g., Baumeister and Leary, 1995) by assuming social roles such as father, brother, friend, colleague, or leader and to then meet their needs for affiliation and intimacy (Murry, 1938; Maslow, 1970). Accordingly, as we indicated previously, self and relatedness are two central components because most personality scholars across eastern and western cultural contexts have proposed them (e.g., Wiggins, 1979; Cheung et al., 1996; Beck, 1999; Yang, 2006; Blatt, 2008). Therefore, the functions of personality, namely, independence and interdependence, should be achieved through two core factors of structure, namely self and relatedness. Furthermore, the development of personality is the process of integration of self with relatedness through the integration of or balance between the functions of independence and interdependence (Erikson, 1968; Caspi and Roberts, 2001).

### Criss-Crossing Self vs. Relatedness and Independence vs. Interdependence

A person must maintain their independence and autonomy, and they must also maintain necessary and appropriate relationships with others—that is, have interdependence. The two major elements of personality, self and relatedness, are the carriers that achieve these two functions: independence and interdependence. Adaptability requires the management of the dynamics of self and others. Of course, the realization of functions is different due to differences in the social and cultural environment of the individual. Therefore, we propose a new two-polarities model of personality with a functional perspective. The most common personality dimensions (or traits) in the dualistic framework proposed in this article are briefly summarized in Table 1.

Corresponding to the two basic dimensions—self and relatedness—there are two types of self—the independent self and interdependent self—and two types of relatedness—intrapersonal relatedness and interpersonal relatedness. These four sub-dimensions commonly perform the basic functions of personality, independence and dependence, where personality is defined as individuals’ differences in behavior or inner process (e.g., Carver and Scheier, 2016).

In this section, we further delve into the two-polarities model we propose and further deconstruct the framework of self and relatedness drawing attention to aspects of existing works of eastern and western personality psychologists. In so doing, our aim is to demonstrate the rationality of the new two-polarities model we propose and show how this model is useful to promote an understanding of personality from a cross-cultural perspective.

#### Underexplored Aspects of the Independent and Interdependent Self

Independent self distinguishes and separates a person from others by autonomy and identity coherence. This self is derived from a belief in the wholeness and uniqueness of each person’s configuration of internal attributes (Waterman, 1981; Sampson, 1988; Murray, 1993; Choi and Kim, 2003). The essential aspect of this view involves a conception of the self as an autonomous and independent person (Markus and Kitayama, 1991). The independent self may exhibit certain ego-defense mechanisms (e.g., depression, rigidity, and impulsiveness) to maintain the individual’s identity (Freedman et al., 1951).

Interdependent self reflects the basic function of maintaining a person’s autonomy or identity by considering the person part of an encompassing social relationship. Social environments, especially other people, serve as a mirror-like reflection to show a person’s uniqueness. This interdependent self has the function of establishing the personality self through association between the person and their social environment. Markus and Kitayama (1991), for example, argued that an individual’s behavior is determined by, contingent on, and, to a large extent, organized by what they perceive to be the thoughts, feelings, and actions of others in their relationships or social context. The interdependent self may employ certain interaction mechanisms to maintain identity (Freedman et al., 1951).

As shown in Table 1, some personality traits defined in previous models can indicate the characteristics of the independent self—for example, neuroticism and openness in the Big Five model (McCrae and Costa, 2008) and novelty, diversity, enterprise, sensation seeking, and life goals in the CPAI model (Fan et al., 2011; Cheung et al., 2013). Some personality traits defined in previous models may indicate the characteristics of the interdependent self—for example, domination (Freedman et al., 1951), face (Zhai, 2013; also in the CPAI model), *lian* (Zhai, 2013), and conscientiousness in the Big Five model (McCrae and Costa, 2008).

Particularly, in terms of Church’s (2000) viewpoint regarding the possibility of integration of trait psychology and cultural psychology, some authors have largely distinguished two types of self in light of the framework of individualist and collectivist cultures. For example, after reviewing the relevant empirical literature about the self in western and eastern contexts, Markus and Kitayama (1991) proposed two types of self-construal, the independent self and interdependent self, in terms of individualist versus collectivist culture. Generally, individuals in an individualist society, which can be represented by the United States, are more likely to embody the independent self because their social environment requires them to embody self-independence. Western cultures emphasize the inherent separateness of distinct people, who must be independent from others and realize and express their unique attributes (Miller, 1988; Markus and Kitayama, 1991). However, eastern cultures, which can be represented by China, emphasize the fundamental connectedness between human beings; thus, individuals in a collectivist society are more interdependent because their environment requires them to maintain interdependence among individuals (Hsu, 1985; Miller, 1988) and to see themselves as part of an encompassing social relationship.

Yang (2004) argued that the Chinese self is expressed in terms of social orientation and individual orientation. In Yang’s (2004) model, the dimension of individual orientation is similar to the construct of the independent self, whereas social orientation is largely similar to the construct of the interdependent self in Markus’ framework. In fact, some other authors have defined two types of self in terms of the cultural differences between east and west in theoretical or empirical studies (e.g., Gao, 1996; Wang and Li, 2003; Mo, 2012). For example, after conducting an experiment, Mo (2012) reported that in addition to having the independent self in the western cultural sense, Chinese people often include family members or close relatives in their self-construct. In a certain sense, this is a manifestation of the independent self and interdependent self in the personality structure.

Derived from a Chinese setting, face has often been defined as a proper reputation and image in social interactions (Cheung et al., 1996). Accordingly, face reflects the interpersonal self to a certain extent. On the basis of an in-depth interpretation of the concept of face, Zhai (2013) introduced the concept of *lian* from the Chinese language. *Lian* further clarifies the interdependent self, showing more details of its function of self establishment in social environment. According to Zhai (2013), *lian* refers to the mind and behavior that an individual expresses after impression management to cater to an image recognized by a certain social circle, whereas face is the sequential position of an image (namely, *lian*) already formed in the minds of people in the social circle or others. The work conducted by Zhai (2013) may indicate that making a deconstructive analysis for interpersonal self is necessary when emphasizing the interpersonal relevance of the personal self in a Chinese context.

In addition, from the broader perspective of social psychology, private versus collective or public selves have been proposed in some other theories, such as the socioanalytic development of personality (Hogan, 1982), self-cognition (Triandis, 1989) collective/group/social identity (Schlenker, 1985; Turner et al., 1987; Brewer and Gardner, 1996), and self-verification (Swann, 1984). Private-self cognition reflects an assessment of the self by the self and includes cognitions involving personal traits, states, and behaviors (Fenigstein et al., 1975; Ybarra and Trafimow, 1998). Similarly, Schlenker (1986) argued that the private self has been afforded prestigious status and is usually regarded as having both structure, containing the organized and relatively stable content of personal experiences, and an active process that guides and regulates thoughts, feelings, and actions. The private self is the core of a person’s inner being and reflects basic, enduring, distinctive, and genuine attributes. Self-reflection and self-identify are the main functions through which the private self acquires, crystallizes, or conveys accurate information on the self. This may be an intrapsychic process of defining automatic and independent individual identity.

By contrast, the collective or public self, which is the self as it is projected in a person’s social life, reflects a process of self-disclosure and self-presentation (Schlenker, 1986) aiming to connect the person with their environment through assessment of the self by a specific reference group or collective. The collective self reflects the self-cognitions based on some collective (Triandis, 1989) because social norms and predilections embed us deeply in a matrix of real and imagined other people who influence our ideas and behaviors (Schlenker, 1986). Therefore, the collective self may reflect the interdependent content of the self.

Furthermore, according to the two-location theory proposed by Trafimow et al. (1991), the private self and the collective self are independent of each other, and the retrieval of a specific type of self-cognition depends on, for example, the individual’s specific cultural setting. People from an individualistic culture may retrieve more private-self cognitions and fewer collective-self cognitions than those from a collectivist culture. However, the private and collective selves could be considered complementary facets of self-identity. They are intertwined and equally significant. As argued by Schlenker (1986), considerable interplay exists between these two selves. The two selves reflect a reciprocal relationship between people’ private self-image and their public projections of self. Self-image influences public behavior, which in turn can modify self-image. Accordingly, both the private self and the collective self are pivotal components of the personality self.

Therefore, in sum, although the independent self-construal versus interdependent self-construal, individual-oriented versus social-oriented self, and private self versus collective self are derived from different theoretical perspectives, they all indicate a dualistic interpretation of personality. The self may not be a one-dimensional construction; it should include the construction of the independent self and that of the interdependent self. Both types of selves are embedded in people’s personality across west and east. It is just that individuals are immersed respectively in individualism or collectivism, which leads to ones’ different representation hierarchy in terms of the two selves.

#### Underexplored Aspects of Intrapersonal Relatedness and Interpersonal Relatedness

Intrapersonal relatedness reflects how an individual thinks about their social world—their social cognition. This type of relatedness indicates how a person relates his/her self to the social environment. Intrapersonal relatedness reflects the relevance of personality by assessing those characteristics expressing how a person associates themselves with their circumstances. As reported in Table 1, some personality traits defined in previously proposed models may indicate the characteristics of intrapersonal relatedness—for example, agreeableness (McCrae and Costa, 2008), and graciousness, defensiveness, self versus social orientation, veraciousness versus slickness, and discipline in the CPAI models (Fan et al., 2011; Cheung et al., 2013).

Interpersonal relatedness defines how a person relates to the social world through behavior or performance, such as social presentation and social transformation (namely, persona). The interpersonal relatedness reflects the relevance of personality by assessing those characteristics expressing how a person associates themselves with circumstances through external behavior and performance. Similarly, some personality traits defined in previously proposed models may indicate characteristics of interpersonal relatedness (also see Table 1) —for example, cooperation (Freedman et al., 1951), extraversion (McCrae and Costa, 2008), and *renqing*, harmony, and interpersonal tolerance in the CPAI models (Fan et al., 2011; Cheung et al., 2013).

In addition, as a second key dimension in the literature on personality (e.g., Wiggins, 1979; McCrae and Costa, 1989), the relatedness construct needs to be explained clearly. Several similar terms have been used in the research on personality, such as relatedness, interpersonal relatedness, and interpersonal personality. The set of terms may have been employed with varying meaning or some overlaps by personality scholars from western and eastern contexts. Two types of relatedness could also be understood in terms of Church’s (2000) viewpoint regarding the possibility of integration of trait psychology and cultural psychology with the framework of individualist and collectivist cultures.

When Blatt and his colleagues proposed their two-polarities personality model, they did not always clearly distinguish relatedness from interpersonal relatedness (e.g., Luyten and Blatt, 2013). However, interpersonal relatedness may not be the whole content of the meaning of relatedness (Fan et al., 2008). Although interpersonal relatedness has been addressed in the models proposed by Blatt (2008) and Cheung et al. (1996, 2001, 2008b), the publications by Blatt and Cheung have never cited one another. Accordingly, the interpersonal relatedness construct may have widely different meanings in their corresponding models, or some overlaps may exist between the interpersonal relatedness considered by Blatt and by Cheung as well as their colleagues. Whether there are two types of interpersonal relatedness must be determined.

According to Blatt (2008) and Luyten and Blatt (2013), interpersonal relatedness refers to reciprocal, meaningful, and personally satisfying interpersonal relationships. Clearly, the interpersonal relatedness defined by Blatt and colleagues mainly reflects one type of external (objective) interpersonal behavior or the corresponding pattern in which a person’s individual differences are expressed through communicated behaviors. This may well represent some western personality psychologists’ understanding of interpersonal relatedness.

As we reviewed previously, the definition of “interpersonal relatedness” by Cheung and her colleagues may consist of two types of relatedness. Furthermore, we empirically examined the data from use of the CPAI-A in a Hong Kong standardization study (Cheung et al., 2008b), mainland China standardization studies (Xie et al., 2016; Dong et al., 2020), and recent investigation conducted in Shanghai, China (Li et al., 2019) as well as the data from use of the CPAI-2 in the original standardization study (Cheung et al., 2008a) and recent data obtained from Chinese college students (Zhou et al., 2021). These data reveal a binary structure of relatedness. One type of interpersonal relatedness is similar to that defined by Blatt and colleagues and mainly manifests as objective or external relatedness; this is measured by *renqing*, harmony, interpersonal tolerance, and social sensitivity subscales. This type of interpersonal relatedness reflects an individual’s persona, social presentation, or social transaction—how the person relates to the social world through actual behavior, expression, or presentation. Therefore, this type of relatedness can be defined as interpersonal relatedness and is similar to the public level of interpersonal personality (Freedman et al., 1951; Leary, 1957), and is assessed by considering the person’s behavior or performance.

Intrapersonal relatedness reflects an individual’s subjective attitude toward relationships with people and intrinsic characteristics related to interpersonal communication. Intrapersonal relatedness is similar to the private level of interpersonal personality (e.g., Freedman et al., 1951; Leary, 1957), and can be assessed through the subject’s descriptions, dreams, values, or other projective outcomes. The concept corresponds to intrapersonal relatedness in the CPAI including graciousness, discipline, thrift, and traditionalism. Intrapersonal relatedness reflects an individual’s social cognition (i.e., how they think about their social world), which is mainly conducted in the mind, hence being termed intrapersonal relatedness.

For another example, although Blatt and Blass (1996) reanalyzed Erikson (1968, 1982) eight-stage linear developmental line together with an additional stage of cooperation versus alienation, trust–mistrust, and cooperation–alienation and intimacy–isolation must not reflect the same type of relatedness. Specifically, trust–mistrust reflects a person’s attitude or cognition regarding the social world (i.e., social cognition), whereas cooperation–alienation and intimacy–isolation reflects how they relate to their social world through specific behaviors and expressions (i.e., persona, social presentation, and social transaction).

## Conclusion and Future Directions

Adopting a cross-cultural perspective across the west and east to understand people’s differences through a concise structure has always been the goal of personality psychologists (e.g., Yang, 2006; Blatt, 2008; Heine and Buchtel, 2009). In this article, we rethink previous work on a broad binary model of self and relatedness and reconstruct the two-polarities personality model comprising relatedness and self. Self and relatedness are the fundamental psychological structure and developmental processes involved in development of the capacity to establish and maintain an integrated personality (Sullivan, 1953; Wiggins, 1979; McCrae and Costa, 1989; Blatt, 2008). The two-dimension model has been theoretically and empirically supported in both western and eastern cultural settings. However, many popular theories (e.g., the Big Five model and Erikson’s model) do not properly distinguish the two basic personality dimensions, although a few attempts have been made (e.g., Digman, 1997; Blatt, 2008). Furthermore, self and relatedness have sometimes been defined differently in different models, particularly those constructed from western versus eastern perspectives.

We argue that the fundamental reason for the aforementioned problems lies in a confusion or failure to distinguish the structure and function of personality; self and relatedness may have various meanings, and different aspects of these dimensions may be highlighted in different cultural settings. Accordingly, we reconstruct the dualistic personality model that includes not only self and relatedness but also independent and interdependent functions. Four sub-dimensions of personality in the dualistic model of self-relatedness are proposed: independent self, interdependent self, intrapersonal relatedness and interpersonal relatedness. Specifically, this integrated model with re-constructed both self and relatedness would advance the field of personality research.

For example, the integrated model is a more complete model of personality unlike other models that emphasize only one dimension. Whether in the western or eastern context, the outcome of our behavior always depends on our overall personality. The only difference is which part plays a greater predictive role. The integrated two-polarities model may have incremental validity above and beyond one dimensional models in predicting individuals’ learning or work performances, and mental health in a specific cultural setting. Additionally, the more complete model should function better when used in cross-cultural studies since some cultures are individualistic and other cultures are collectivistic.

In addition, even within a single country, there are cultural subgroups (e.g., racial ethnic minorities in the United States), this more complete model may provide greater cross-cultural/cross-ethnic validity. For example, because the differences between an anaclitic/sociotropic depression and an introjective/autonomous depression are congruent with predictions made by the traditional two-polarities personality model of self and relatedness (e.g., Luyten and Blatt, 2013), our new-proposed two-polarities model, which further subdivides self and relationships, may help people to understand, predict and even cope with depression more accurately in different cultural backgrounds.

However, we only preliminarily reconstruct the self-relatedness personality framework, and some important issues remain to be investigated in future works. First, the validity of the theory and practice of the dualistic model proposed in this article must be examined. Although we provide a brief summary of personality dimensions and traits by discussing the major models of personality (e.g., the Big Five model, Erikson’s personality development stages and tasks, and the CPAI models), some other key models of personality [e.g., Murry’s (1938) or Maslow’s (1970) need model, and Freud’s (1962) personality structure and development model] must be further reconstructed in the framework of self-relatedness. Second, more strong theoretical and empirical evidence is required to support the proposed self-relatedness dualistic model. Some other authors (e.g., Digman, 1997; Blatt, 2008; Luyten and Blatt, 2013) have favorably reviewed relevant works. However, our proposed model is congruent with a broad range of theoretical formulations regarding personality development, personality structure, personality functions, personality assessment, and even the neurophysiological mechanism and biological genetic basis of personality.

Third, if a model is useful for understanding personality and the validation of the model in predicting people’s behavior or performance, it largely depends on reliable and validated measurement and assessment practices of the personality construct under the corresponding framework. Accordingly, how to operationalize the constructs such as independent/interdependent self and intrapersonal/interpersonal relatedness in this model and develop corresponding reliable and effective evaluation tools are also important issues that we need to solve in the future. These assessment tools can not only clearly reflect the basic meaning of those key constructs that we proposed, but also avoid different cultural prejudices and achieve cross-cultural invariance. Some specific issues should be well solved in practice. Furthermore, for dealing with those measurement-related issues, some other questions have also been answered theoretically and practically. For example, theoretically, peoples within both western and eastern cultures show greater collectivist or individualist tendencies, respectively. From a functionalist perspective, what does it mean to have a greater interdependent self and interpersonal relatedness in a more individualistic culture? Or what does it mean to have a greater independent self and intrapersonal relatedness in a more collectivistic culture? In the framework of self-relatedness personality with the functions of independence and interdependence, will the cultural aggregate norms of personality have any reflection in a specific cultural setting or ideology? And how to implement these ideas or assumptions should be well examined in the future work.

Fourth, the association between eastern versus western culture and the self-relatedness personality model should be further explored. Although we have attempted to combine personality dimensions derived from different cultural settings in the dualistic model, considerable working space remains in this domain. For example, Zhai (2013) defined the construct *lian* in terms of the popular term face on the basis of empirical evidence, but our model regards both *lian* and face as part of the interpersonal self. Clearly, as reported by Yang (2006), some personality attributes may be relatively fixed in each type of culture (e.g., harmony in Chinese culture and openness in American culture) whereas others may be relatively malleable (e.g., extroversion in Chinese culture) though opposite patterns of relative fixedness and malleability. Specifically, the self and relevant traits or functions may be more powerful, pervasive, and influential among western people, whereas relatedness and relevant traits may be more powerful, pervasive, and influential among eastern people.

All in all, we believe that the proposed two polarities model will advance the integration perspective of studying personality across western and eastern cultures. At the same time, we also hope that this two polarities model can help scholars account for differences in personality between eastern and western cultures in the context of globalization and glocalization by comprehensively considering the structure and function of personality for people in a specific social context.

## Author Contributions

All authors listed have made a substantial, direct, and intellectual contribution to the work, and approved it for publication.

## Conflict of Interest

The authors declare that the research was conducted in the absence of any commercial or financial relationships that could be construed as a potential conflict of interest.

## Publisher’s Note

All claims expressed in this article are solely those of the authors and do not necessarily represent those of their affiliated organizations, or those of the publisher, the editors and the reviewers. Any product that may be evaluated in this article, or claim that may be made by its manufacturer, is not guaranteed or endorsed by the publisher.
